# Association between Socioeconomic Status and Digestive Tract Cancers: A Case-Control Study

**DOI:** 10.3390/cancers12113258

**Published:** 2020-11-04

**Authors:** Yukino Kawakatsu, Yuriko N. Koyanagi, Isao Oze, Yumiko Kasugai, Hisayoshi Morioka, Rui Yamaguchi, Hidemi Ito, Keitaro Matsuo

**Affiliations:** 1 Division of Cancer Epidemiology and Prevention, Aichi Cancer Center, Nagoya 464-8681, Japan; y.kawakatsu@aichi-cc.jp (Y.K.); i_oze@aichi-cc.jp (I.O.); ymaeda@aichi-cc.jp (Y.K.); 2 Department of Public Health, Tokushima University Graduate School of Biomedical Sciences, Tokushima 770-8501, Japan; hisayoshi.morioka@tokushima-u.ac.jp; 3 Division of Cancer Information and Control, Aichi Cancer Center, Nagoya 464-8681, Japan; ykoyanagi@aichi-cc.jp (Y.N.K.); hidemi@aichi-cc.jp (H.I.); 4 Division of Cancer Systems Biology, Aichi Cancer Center, Nagoya 464-8681, Japan; r.yamaguchi@aichi-cc.jp; 5 Department of Cancer Informatics, Nagoya University Graduate School of Medicine, Nagoya 466-8550, Japan; 6 Department of Descriptive Cancer Epidemiology, Nagoya University Graduate School of Medicine, Nagoya 466-8550, Japan; 7 Department of Cancer Epidemiology, Nagoya University Graduate School of Medicine, Nagoya 466-8550, Japan

**Keywords:** socioeconomic status, digestive tract cancer, cancer risk

## Abstract

**Simple Summary:**

An association between socioeconomic status (SES) and cancer risk has been reported, but little is known in Asia. We revealed an association between SES, including education level and areal deprivation index (ADI), and digestive tract cancers in Japan. Lower SES was associated with an increased risk of digestive cancers. For stomach cancer, the positive association with ADI disappeared following an additional adjustment of *Helicobacter pylori* infection and/or atrophic gastritis status. Cancer prevention policy should consider both individual and regional perspectives by the integration of SES in the target population.

**Abstract:**

Although socioeconomic status (SES) has been associated with cancer risk, little research on this association has been done in Japan. To evaluate the association between SES and digestive tract cancer risk, we conducted a case-control study for head and neck, esophageal, stomach, and colorectal cancers in 3188 cases and the same number of age- and sex-matched controls within the framework of the Hospital-based Epidemiological Research Program at Aichi Cancer Center III (HERPACC III). We employed the education level and areal deprivation index (ADI) as SES indicators. The association was evaluated with odds ratios (ORs) and 95% confidence intervals (CIs) by conditional logistic models adjusted for potential confounders. Even after allowance for known cancer risk factors, the education level showed linear inverse associations with head and neck, stomach, and colorectal cancers. Compared to those educated to junior high school, those with higher education showed statistically significantly lower risks of cancer (0.43 (95% CI: 0.27–0.68) for head and neck, 0.52 (0.38–0.69) for stomach, and 0.52 (0.38–0.71) for colorectum). Consistent with these results for the educational level, the ADI in quintiles showed positive associations with head and neck, esophageal, and stomach cancers (*p*-trend: *p* = 0.035 for head and neck, *p* = 0.02 for esophagus, and *p* = 0.013 for stomach). Interestingly, the positive association between ADI and stomach cancer risk disappeared in the additional adjustment for *Helicobacter pylori* infection and/or atrophic gastritis status. In conclusion, a lower SES was associated with an increased risk of digestive cancers in Japan and should be considered in cancer prevention policies for the target population.

## 1. Introduction

Digestive tract cancers are among the most common tumor types, affecting over 4.1 million people worldwide and causing 2.6 million deaths in 2018 [1]. They are particularly common in Asia [1,2]. Reducing the adverse health impacts of these cancers will require the accumulation of knowledge that leads to feasible preventive actions. Numerous epidemiologic studies have been conducted, and associations with modifiable risk factors have been identified, such as smoking or alcohol drinking [3,4,5,6,7,8]. Recently, an association with socioeconomic status (SES) was reported in Western countries [9,10,11,12]. However, the interpretation of this new evidence is complicated by the correlation between SES and these modifiable risk factors [13,14]. Therefore, there is a need for research that can disentangle the effects of these socioeconomic factors and preventable factors on digestive tract cancer risks. Given that a variety of individual exposures may have common socioeconomic causes at the population level, it is important to discuss cancer prevention from both an individual and regional perspective, with a particular focus on lower SES regions. To date, however, few studies have examined the association between socioeconomic differences and cancer risks in Japan.

To evaluate the association between the risk of digestive tract cancers, including head and neck, esophageal, stomach, and colorectal cancers, and SES with regard to the levels of individual education and neighborhood deprivation, we conducted a case-control study to examine the association with consideration to known risk factors.

## 2. Results

Table 1 shows the background characteristics of cases and matched controls by the four sites of cancer. Age and sex were appropriately matched. Heavier alcohol consumption was more prevalent in all sites of cancer compared with the matched controls, especially for esophageal cancer (51.1% in cases, and 13.5% in controls). Current smoking was more prevalent in the cases, and cumulative exposure to cigarettes was clearly higher in the cases (38.5% in cases and 23.5% in controls for head and neck, 46.5% in cases and 23.3% in controls for esophagus, 30.3% in cases and 22.6% in controls for stomach, and 22.7% in cases and 20.4% in controls for colorectal). The body mass index (BMI) was lower in the cases than controls in head and neck (percentage of underweight: 14.7% in cases and 7.0% in controls), esophageal (15.5% in cases and 3.0% in controls), and stomach cancers (10.4% in cases and 6.3% in controls). We observed no remarkable difference in BMI for colorectal cancer. Diabetes was significantly more common in the cases than controls for head and neck cancer (12.6% in cases and 10.1% in controls) and was significantly more common in the controls than cases for esophageal cancer (7.4% in cases and 12.5% in controls) but not for the other types of cancer. A family history of the subject cancer was common in stomach cancer (26.3% in cases and 17.6% in controls) but not in the other cancers. Regarding physical activity, lower activity was more common in cases for all types of cancers. Vegetable/fruit intakes were lower in the cases than in controls, except for esophageal cancer. No clear difference in the intake of meat and processed meat was observed between the cases and controls across all sites.

Table 2 shows the association between educational level and risk of cancer by site in multivariable conditional logistic regression models. In the crude models, we observed a linear inverse association between educational level and risk of cancer for all sites of cancer. We explored two multivariable models and observed a consistent linear inverse association in a crude analysis in all sites of cancer except esophageal cancer (*p*-trend in Model 2: *p* = 1.4 × 10^−4^ for head and neck, *p* = 0.172 for esophagus, *p* = 5.5 × 10^−7^ for stomach, and *p* = 1.2 × 10^−5^ for colorectum). Compared to participants with education to junior high school, those with higher education showed a statistically significantly lower risk of cancer (0.43 (95% CI: 0.27–0.68) for head and neck, 0.52 (0.38–0.69) for stomach, and 0.52 (0.38–0.71) for colorectum). We did not observe a statistically significant association with esophageal cancer; however, the point estimates for higher education and moderate education levels for esophageal cancer were 0.64 and 0.63, respectively, indicating a modest but consistent inverse association between educational level and risk. To further explore the possible heterogeneity of this association by age, we conducted an additional stratified analysis by age group for Model 2 (Table S3). We did not observe a clear heterogeneity between younger and older groups.

Table 3 shows the association between the deprivation index and risk of each cancer by site. In the crude model, we observed a statistically significant or marginally significant trend in all sites of cancer. Consistent with the crude model, the two multivariable models also showed positive associations with head and neck, esophageal, and stomach cancers (*p* for trends of 0.035, 0.02, and 0.013 for head and neck, esophagus, and stomach, respectively). In contrast, the positive association observed in the crude model for colorectal cancer disappeared after an adjustment for confounders. Of note, a statistically higher risk for stomach cancer was seen in all quintile levels except Q1 as reference.

Table S1 shows that the association between education level and stomach cancer was consistent regardless of *Helicobacter pylori* infection and atrophic gastritis status. Table S2 shows that, after adjustment for *H. pylori* and atrophic gastritis status, the association between the deprivation index and stomach cancer risk was attenuated. Although we observed significant linear trends in the crude and two multivariable analyses for overall stomach cancer (Table 3), we did not see any significant trend in the analyses that considered *H. pylori* infection and atrophic gastritis status.

## 3. Discussion

In this case–control study, we identified an association between SES and digestive tract cancer risks in 3188 cases and the same number of age- and sex-matched controls. Despite adjustments for known cancer risk factors, the education level showed a linear inverse association with head and neck, stomach, and colorectal cancers, while the areal deprivation index (ADI) showed a positive association with head and neck, esophageal, and stomach cancers. This association between poor SES and a higher risk of digestive tract cancer was accordingly consistent across two different measures.

Previous studies have indicated an inverse association between SES and cancer risk [9,10,12]. Among the digestive tract cancers, inverse associations have been reported for head and neck, esophageal, and stomach cancers [12,15,16]. For colorectal cancer, in contrast, both positive and inverse associations have been reported [11,17,18], with lower SES associated with an increased incidence of colorectal cancer in the United States versus an inverse association among Europeans [17]. A population-based cohort study in Japan showed that the risk of colorectal cancer incidence was lower in men and women with a higher neighborhood deprivation index than in those in lower index categories [19]. In China, positive associations were seen for per capita GDP and disposable income for area-level SES, household income, and the number of assets for individual-level SES; in contrast, however, education showed no association with colorectal cancer [18]. In the present study, we found an inverse association with all sites of digestive tract cancer. This discrepancy may be explained by differences in the associations between SES indicators and lifestyle risk factors or cancer screening rates across countries and times. The reasons for this across-study or -regional difference is not clear but is likely explained in part by social and environmental differences.

Eliminating health disparities will require attention to all SES components and the pathways by which they influence health [20]. Redressing fundamental economic and social inequality is no simple matter. In general, we need to educate people about risk reduction, especially via modifiable risk factors. Furthermore, in this study, disparities due to SES exist even after adjusting for known risk factors. Therefore, it is desirable to conduct further researches to reveal hidden factors behind SES and cancer.

A unique finding of this study was that the positive association between ADI and stomach cancer risk disappeared following the additional adjustment for *H. pylori* infection and/or atrophic gastritis status. Given that previous studies have shown associations between SES and *H. pylori*/atrophic gastritis [21], we speculate that SES may be a strong surrogate for *H. pylori*/atrophic gastritis status. Supporting this, a similar phenomenon was reported in a prospective study conducted in Europe: a significant inverse association between the higher educational level and risk of gastric cancer disappeared after an adjustment for *H. pylori* seroprevalence [16]. In our study, however, we observed this phenomenon with ADI only and not with the education level. This difference between Europe and Japan might be attributable to differences in social context between the ADI and education level between the two areas. To prevent against stomach cancers in those with low SES, interventions targeting *H. pylori* infection such as eradication or prevention may be effective. Although the way *H. pylori* is transmitted remains still not fully clear, the level of contamination is strongly dependent on the familial and environmental context, with a drastic impact of living conditions with poor hygiene and sanitation [22]. According to many epidemiologic studies, water could be an important source of *H. pylori* contamination [23]. Improving water and sewerage systems, especially in rural area, is likely to reduce the risk of *H. pylori* infection for low SES. This possibility warrants further evaluation.

The strengths of this study include its large number of participants and case-control design, with consideration for the relevant confounding variables, including *H. pylori* infection. Both the education level and ADI showed an association with digestive tract cancers, with consideration of the known risk factors. Given that the ADI does not include the educational level in its estimation, the consistency of our results across the two measures supports the robustness of our findings. Among the limitations, the study used a hospital-based case-control design that might have had a degree of selection bias. However, subjects were recruited before the diagnosis of cancer or noncancer and before we had knowledge of their ADI and educational levels, suggesting that this bias was unlikely. Second, it is difficult to completely avoid a degree of information bias, as lifestyle factors were collected via a self-reported questionnaire. We minimized this possibility by asking about lifestyle factors in the year before onset of disease. Educational levels are less likely to be biased due to the timing of the questionnaire. Since the ADI of areas with a small number of households are considered statistically unstable [24], we conducted a sensitivity analysis by excluding areas with a small number of households and confirmed that similar results were obtained (Table S4). Finally, this study was a single hospital-based study, and accessibility to our hospital might have caused a degree of bias by case status. However, most of our cases and controls were from the Nagoya metropolitan area, and public and private transportation to the hospital are widely available. In addition, as shown in Figures S1 and S2, the physical distances between the residential addresses and our hospital did not differ by either the ADI or educational level. This bias therefore seems unlikely.

## 4. Materials and Methods

### 4.1. Study Population

All first-visit outpatients at the Aichi Cancer Center Hospital (ACCH) between December 2005 and March 2013 were asked to participate in the Hospital-based Epidemiologic Research Program at Aichi Cancer Center (HERPACC)-3. The framework of HERPACC has been described elsewhere [25]. We conducted a case-control study for each eligible type of cancer among the participants of HERPACC-3. Cases were incident cases of 587 head and neck cancer, 503 esophageal cancer, 1146 stomach cancer, and 952 colorectal cancer. Controls were randomly selected from among HERPACC-3 participants who were confirmed to have no cancer or history of neoplasm and individually matched with cases by age (±5 years) and sex at a case-control ratio of 1:1. We defined noncancer first-visit outpatients as a population in which cases may arise, under the assumption that they will likely visit the ACCH if they develop cancer in the future [25]. All participants gave written informed consent to participate, and 66.4% of participants responded to a self-administered questionnaire, and 62% of participants provided a peripheral blood sample [26]. The study was approved by the Institutional Ethics Committee at Aichi Cancer Center (approval number: ACC-2019-2-33).

### 4.2. Site Classification

The following International Classification of Diseases for Oncology, Third Edition (ICD-O-3) codes were used [27]: head and neck cancer, C00–C06, C09–C14, and C30–C32; esophageal cancer, C15; stomach cancer, C16; and colorectal cancer, C18–C20.

### 4.3. Evaluation of Socioeconomic Status

Information on educational levels was collected from first-visit outpatients using a self-administered questionnaire. Educational status was classified into the three categories of junior high school, high school, and higher education.

Regarding deprivation, we used the areal deprivation index (ADI), which consists of weighted sums of a number of census-based variables. The concept and procedures for the Japanese deprivation index are detailed elsewhere [24,28]. We calculated ADI at the “Cho-Aza (CA)” level—the smallest administrative unit provided for in the index—using data from the 2005 Census of Japan. We categorized the deprivation level into five groups by quintiles of the participants’ deprivation index values.

For calculations, residence was considered the residential address at the time of enrollment. This was geocoded to identify living areas in CA units of the 2005 Census of Japan. We excluded 15 participants whose addresses could not be geocoded and 8 whose census information regarding CA was not provided by the statistical bureau [24].

### 4.4. Evaluation of Environmental Factors

Information on the environmental risk factors was collected using a self-administered questionnaire. At first-visit to our hospital, each participant was asked about their lifestyle before the development of the symptoms that made them visit the hospital. As detailed elsewhere [29], daily alcohol intake (g/day) was used as a measure of drinking intensity and calculated using information on the frequency of alcohol drinking and the total amount of pure alcohol consumed during each drinking session. Alcohol consumption was classified into the four groups of never, low (0–23 g/day), moderate (23–46 g/day), and heavy (≥46 g/day). Smoking status was classified into the three groups of never, former, and current. Smoking dose was evaluated as pack-years (PY), calculated by multiplying the number of packs consumed per day by the number of years of smoking. Participants were categorized into the five groups of never, PY <20, PY <40, PY <60, and PY 60 or more. Body mass index (BMI) was calculated as the weight in kilograms divided by the height in meters squared and divided into the four groups of <18.5, 18.5–21.9, 22–24.9, and ≥25.0. History of diabetes and family history of each cancer were obtained as “yes” or “no”. Physical activity was evaluated as metabolic equivalent (MET) hours per week [30], calculated by the frequency, intensity, and amount of time per session and classified into the four groups of never, 0–10, 10–20, and ≥20 MET hours per week. Energy-adjusted fruit/vegetable intake was estimated by the residual method [31,32] using information from a validated food frequency questionnaire [33]. For fruit/vegetable intake and total energy intake, participants were classified into four groups according to the distributions of the respective factors among the controls (quartiles). The frequencies of meat (beef or pork) intake and processed meat intake were classified into the three categories of <1, 1–4, and ≥5 times/week. For *H. pylori* infection status, plasma or serum IgG (immunoglobulin G) antibody levels for *H. pylori* were measured using a commercially available direct enzyme-linked immunosorbent assay kit (E Plate “Eiken” H. pylori Antibody; Eiken Kagaku, Tokyo, Japan), with a positive infection status defined as anti-*H. pylori* IgG >10 U/mL. For atrophic gastritis status, plasma/serum pepsinogens (PGs) were measured by chemiluminescence enzyme immunoassay, with a positive gastric mucosal atrophy defined as PG I ≤70 ng/mL and PG I/PG II ≤3.

### 4.5. Statistical Analysis

Differences in the distribution of risk factors between cases and controls were evaluated using the χ^2^ test. The exposure of interest in this study was SES, for which we used educational level and ADI in quintiles as indices. To evaluate the association between SES and risk of each cancer, we estimated the odds ratios (OR) and corresponding 95% confidence intervals (CIs) by the conditional logistic regression models. We used three models: a crude model and Model 1 and Model 2. The crude model was age- and sex-matched; Model 1 was a multivariable model that adjusted for alcohol consumption (never, 0–23 g, 23–46 g, and ≥46 g ethanol/day); pack-years (0, <20, 20–40, and ≥40 PYs); and family history of each cancer (yes/no). Model 2 further adjusted for BMI (kg/m^2^); history of diabetes (yes/no); physical activity (0, 0–10, 10–20, and ≥20 MET hours/week); fruit/vegetable intake (quartile); total energy intake (kcal/day); frequency of meat intake (<1, 1–4, and ≥5 times/week); frequency of processed meat intake (<1, 1–4, and ≥5 times/week); and family history of each cancer (yes/no), in addition to the items adjusted for in Model 1. As *H. pylori* infection is a well-established risk factor for stomach cancer, with a well-known correlation with socioeconomic status [22,34], to estimate the effect of *H. pylori* infection in our evaluation of SES on cancer risk, we evaluated multivariable models that added the *H. pylori* status (positive or negative) and gastric atrophy (positive or negative) to subgroups with available blood samples within each of the three models.

All statistical analyses were carried out using Stata version 15 (Stata Corporation, College Station, TX, USA). Two-sided *p*-values < 0.05 were considered to show statistical significance.

## 5. Conclusions

We found an association between SES and digestive tract cancers. With regard to stomach cancer, however, the association with ADI was attenuated after adjustment for the *H. pylori*/atrophic gastritis status. Cancer prevention policy should consider both individual and regional perspectives by the integration of SES in the target population.

## Figures and Tables

**Table 1:** Subject characteristics.

Characteristics	Head and Neck				Esophagus				Stomach				Colorectal			
	Cases		Controls		Cases		Controls		Cases		Controls		Cases		Controls	
	(*n* = 587)	%	(*n* = 587)	%	(*n* = 503)	%	(*n* = 503)	%	(*n* = 1146)	%	(*n* = 1146)	%	(*n* = 952)	%	(*n* = 952)	%
Sex																
Male	442	75.3%	442	75.3%	437	86.9%	437	86.9%	827	72.2%	827	72.2%	586	61.6%	586	61.6%
Female	145	24.7%	145	24.7%	66	13.1%	66	13.1%	319	27.8%	319	27.8%	366	38.4%	366	38.4%
Age																
<40	62	10.6%	58	9.9%	1	0.2%	4	0.8%	54	4.7%	55	4.8%	42	4.4%	50	5.3%
40–49	54	9.2%	62	10.6%	23	4.6%	24	4.8%	82	7.2%	106	9.2%	108	11.3%	113	11.9%
50–59	138	23.5%	142	24.2%	134	26.6%	138	27.4%	296	25.8%	290	25.3%	265	27.8%	262	27.5%
60–69	216	36.8%	203	34.6%	242	48.1%	215	42.7%	452	39.4%	429	37.4%	351	36.9%	345	36.2%
>70	117	19.9%	122	20.8%	103	20.5%	122	24.3%	262	22.9%	266	23.2%	186	19.5%	182	19.1%
Mean age (SD)	59.3 (12.3)		59 (12.4)		63.3 (7.7)		63.0 (8.4)		61.3 (10.4)		60.9 (10.9)		60.1 (10.5)		59.75 (11.0)	
Alcohol consumption																
Never	184	31.3%	215	36.6%	51	10.1%	157	31.2%	436	38.0%	417	36.4%	376	39.5%	401	42.1%
Low	138	23.5%	204	34.8%	64	12.7%	184	36.6%	339	29.6%	417	36.4%	298	31.3%	331	34.8%
Mod	86	14.7%	87	14.8%	121	24.1%	90	17.9%	170	14.8%	159	13.9%	109	11.4%	109	11.4%
Heavy	171	29.1%	74	12.6%	257	51.1%	68	13.5%	186	16.2%	143	12.5%	161	16.9%	98	10.3%
Unknown	8	1.4%	7	1.2%	10	2.0%	4	0.8%	15	1.3%	10	0.9%	8	0.8%	13	1.4%
Smoking status																
Never	166	28.3%	237	40.4%	69	13.7%	176	35.0%	413	36.0%	483	42.1%	430	45.2%	470	49.4%
Former	192	32.7%	211	35.9%	199	39.6%	208	41.4%	381	33.2%	400	34.9%	302	31.7%	284	29.8%
Current	225	38.3%	138	23.5%	234	46.5%	117	23.3%	347	30.3%	259	22.6%	216	22.7%	194	20.4%
Unknown	4	0.7%	1	0.2%	1	0.2%	2	0.4%	5	0.4%	4	0.3%	4	0.4%	4	0.4%
Pack years																
Never	168	28.6%	239	40.7%	70	13.9%	178	35.4%	414	36.1%	483	42.1%	433	45.5%	473	49.7%
0 ≤ PY < 20	85	14.5%	120	20.4%	59	11.7%	78	15.5%	160	14.0%	190	16.6%	145	15.2%	163	17.1%
20 ≤ PY < 40	125	21.3%	103	17.5%	131	26.0%	110	21.9%	224	19.5%	218	19.0%	179	18.8%	141	14.8%
40 ≤ PY < 60	109	18.6%	65	11.1%	138	27.4%	74	14.7%	193	16.8%	135	11.8%	109	11.4%	87	9.1%
60 ≤ PY	77	13.1%	46	7.8%	88	17.5%	44	8.7%	111	9.7%	83	7.2%	68	7.1%	55	5.8%
Unknown	23	3.9%	14	2.4%	17	3.4%	19	3.8%	44	3.8%	37	3.2%	18	1.9%	33	3.5%
BMI (body mass index)																
BMI < 18.5	86	14.7%	41	7.0%	78	15.5%	15	3.0%	119	10.4%	72	6.3%	66	6.9%	51	5.4%
18.5 ≤ BMI < 25	390	66.4%	423	72.1%	381	75.7%	368	73.2%	850	74.2%	821	71.6%	677	71.1%	690	72.5%
25 ≤ BMI < 30	100	17.0%	106	18.1%	40	8.0%	104	20.7%	157	13.7%	224	19.5%	182	19.1%	185	19.4%
30 ≤ BMI < 35	5	0.9%	13	2.2%	1	0.2%	10	2.0%	16	1.4%	22	1.9%	23	2.4%	16	1.7%
35 ≤ BMI < 40	2	0.3%	0	0.0%	0	0.0%	1	0.2%	0	0.0%	1	0.1%	1	0.1%	3	0.3%
40 ≤ BMI < 45	0	0.0%	0	0.0%	0	0.0%	0	0.0%	0	0.0%	1	0.1%	1	0.1%	0	0.0%
Unknown	4	0.7%	4	0.7%	3	0.6%	5	1.0%	4	0.3%	5	0.4%	2	0.2%	7	0.7%
Diabetes																
Yes	74	12.6%	59	10.1%	37	7.4%	63	12.5%	111	9.7%	131	11.4%	106	11.1%	89	9.3%
No	508	86.5%	528	89.9%	459	91.3%	440	87.5%	1028	89.7%	1012	88.3%	836	87.8%	860	90.3%
Unknown	5	0.9%	0	0.0%	7	1.4%	0	0	7	0.6%	3	0.3%	10	1.1%	3	0.3%
Family history of each cancer																
Yes	19	3.2%	11	1.9%	16	3.2%	9	1.8%	301	26.3%	202	17.6%	135	14.2%	117	12.3%
No	568	96.8%	576	98.1%	487	96.8%	494	98.2%	845	73.7%	944	82.4%	817	85.8%	835	87.7%
Physical activity (metabolic equivalent (MET)-hour per week)																
0	199	33.9%	107	18.2%	125	24.9%	76	15.1%	268	23.4%	204	17.8%	223	23.4%	185	19.4%
0 < MET-hours < 10	223	38.0%	289	49.2%	207	41.2%	232	46.1%	488	42.6%	527	46.0%	428	45.0%	453	47.6%
10 ≤ MET-hours < 20	74	12.6%	90	15.3%	80	15.9%	87	17.3%	188	16.4%	187	16.3%	155	16.3%	147	15.4%
20 ≤ MET-hours	74	12.6%	94	16.0%	81	16.1%	94	18.7%	178	15.5%	201	17.5%	126	13.2%	142	14.9%
Unknown	17	2.9%	7	1.2%	10	2.0%	14	2.8%	24	2.1%	27	2.4%	20	2.1%	25	2.6%
Vegetable/fruit intake																
1st quartile	210	35.8%	147	25.0%	157	31.2%	126	25.0%	361	31.5%	287	25.0%	260	27.3%	238	25.0%
2nd quartile	127	21.6%	147	25.0%	123	24.5%	126	25.0%	237	20.7%	286	25.0%	243	25.5%	238	25.0%
3rd quartile	127	21.6%	147	25.0%	123	24.5%	126	25.0%	277	24.2%	287	25.0%	263	27.6%	238	25.0%
4th quartile	123	21.0%	146	24.9%	100	19.9%	125	24.9%	271	23.6%	286	25.0%	186	19.5%	238	25.0%
Beef/pork intake																
1/week	132	22.5%	132	22.5%	110	21.9%	112	22.3%	266	23.2%	266	23.2%	208	21.8%	193	20.3%
1–4/week	417	71.0%	421	71.7%	373	74.2%	368	73.2%	829	72.3%	814	71.0%	677	71.1%	704	73.9%
5≤/week	21	3.6%	28	4.8%	14	2.8%	19	3.8%	40	3.5%	60	5.2%	55	5.8%	46	4.8%
Unknown	17	2.9%	6	1.0%	6	1.2%	4	0.8%	11	1.0%	6	0.5%	12	1.3%	9	0.9%
Processed meat intake																
1/week	266	45.3%	278	47.4%	251	49.9%	263	52.3%	571	49.8%	597	52.1%	453	47.6%	452	47.5%
1–4/week	274	46.7%	259	44.1%	210	41.7%	206	41.0%	496	43.3%	465	40.6%	414	43.5%	434	45.6%
5≤/week	29	4.9%	40	6.8%	33	6.6%	29	5.8%	69	6.0%	74	6.5%	72	7.6%	55	5.8%
Unknown	18	3.1%	10	1.7%	9	1.8%	5	1.0%	10	0.9%	10	0.9%	13	1.4%	11	1.2%
*Helicobacter pylori* IgG (immunoglobulin G) test																
Positive									570	49.7%	333	29.1%				
Negative									225	19.6%	462	40.3%				
Unmeasured									351	30.6%	351	30.6%				
Atrophic gastritis defined by pepsinogen testing																
Positive									369	32.2%	158	13.8%				
Negative									426	37.2%	637	55.6%				
Unmeasured									351	30.6%	351	30.6%				

**Table 2:** Association between educational level and risk of subject cancers by site ^4^.

Educational Level	Case	Control	Crude ^1^				Model 1 ^2^				Model 2 ^3^			
			Odds Ratio	(95% Conf. Interval)		*p* > z	Odds Ratio	(95% Conf. Interval)		*p* > z	Odds Ratio	(95% Conf. Interval)		*p* > z
Head and Neck														
≤junior high school	123	68	1	Reference			1	Reference			1	Reference		
≤high school	228	215	0.54	0.37	0.78	0.001	0.62	0.42	0.94	0.023	0.67	0.43	1.03	0.069
higher education	232	297	0.37	0.26	0.55	4.2 × 10^−7^	0.43	0.29	0.66	9.0 × 10^−5^	0.43	0.27	0.68	3.2 × 10^−4^
					trend *p* =	3.0 × 10^−7^			trend *p* =	3.6 × 10^−5^			trend *p* =	1.4 × 10^−4^
Esophagus														
≤junior high school	103	67	1	Reference			1	Reference			1	Reference		
≤high school	193	175	0.70	0.47	1.02	0.063	0.76	0.46	1.25	0.278	0.63	0.35	1.13	0.121
higher education	202	254	0.51	0.35	0.74	3.8 × 10^−4^	0.71	0.43	1.17	0.175	0.64	0.36	1.15	0.137
					trend *p* =	2.7 × 10^−4^			trend *p* =	0.181			trend *p* =	0.172
Stomach														
≤junior high school	193	138	1	Reference			1	Reference			1	Reference		
≤high school	481	415	0.80	0.61	1.03	0.088	0.79	0.61	1.04	0.089	0.79	0.60	1.05	0.105
higher education	453	583	0.50	0.38	0.65	4.3 × 10^−7^	0.52	0.39	0.69	5.5 × 10^−6^	0.52	0.38	0.69	9.9 × 10^−6^
					trend *p* =	8.4 × 10^−9^			trend *p* =	2.8 × 10^−7^			trend *p* =	5.5 × 10^−7^
Colorectum														
≤junior high school	166	107	1	Reference			1	Reference			1	Reference		
≤high school	386	350	0.70	0.53	0.94	0.016	0.70	0.52	0.93	0.016	0.69	0.51	0.93	0.016
higher education	390	484	0.50	0.37	0.67	2.3 × 10^−6^	0.51	0.38	0.69	1.1 × 10^−5^	0.52	0.38	0.71	2.9 × 10^−5^
					trend *p* =	3.5 × 10^−7^			trend *p* =	2.6 × 10^−6^			trend *p* =	1.2 × 10^−5^

^1^ Crude model considering matching factors (age and sex) in the conditional logistic regression model. ^2^ Model 1 further adjusted for alcohol intake, pack-years (PY) of smoking, and family history of subject cancer. ^3^ Model 2 further adjusted for BMI, past history of diabetes, physical activity (metabolic equivalent (MET)-hour per week), vegetable/fruit intake, beef/pork intake, and processed meat intake. ^4^ Some cases were excluded because the education status was unknown or other (11 cases of head and neck cancer, 12 cases of esophageal cancer, 29 cases of stomach cancer, and 21 cases of colorectal cancer were excluded).

**Table 3:** Association between quintiles of the deprivation index and risk of subject cancers by sites ^4^.

Quintile of ADI	Case	Control	Crude ^1^				Model 1 ^2^				Model 2 ^3^			
			Odds Ratio	(95% Conf. Interval)		*p* > z	Odds Ratio	(95% Conf. Interval)		*p* > z	Odds Ratio	(95% Conf. Interval)		*p* > z
Head and Neck														
Q1	97	117	1	Reference			1	Reference			1	Reference		
Q2	110	117	1.16	0.79	1.71	0.457	1.37	0.89	2.12	0.151	1.35	0.84	2.16	0.214
Q3	116	117	1.22	0.83	1.78	0.309	1.22	0.80	1.87	0.352	1.07	0.67	1.69	0.784
Q4	133	117	1.40	0.95	2.04	0.086	1.63	1.07	2.49	0.023	1.62	1.03	2.56	0.039
Q5	129	117	1.34	0.92	1.94	0.123	1.52	1.00	2.30	0.048	1.59	1.02	2.48	0.041
					trend *p* =	0.085			trend *p* =	0.047			trend *p* =	0.035
Esophagus														
Q1	71	100	1	Reference			1	Reference			1	Reference		
Q2	67	101	0.95	0.61	1.47	0.806	0.75	0.41	1.38	0.352	0.72	0.36	1.44	0.357
Q3	97	99	1.41	0.93	2.14	0.108	1.36	0.79	2.35	0.265	1.50	0.80	2.84	0.208
Q4	130	100	1.82	1.22	2.73	0.004	1.27	0.75	2.16	0.378	1.22	0.66	2.23	0.525
Q5	134	100	1.88	1.25	2.84	0.003	1.48	0.85	2.58	0.162	1.77	0.94	3.35	0.078
					trend *p* =	4.7 × 10^−5^			trend *p* =	0.038			trend *p* =	0.021
Stomach														
Q1	156	230	1	Reference			1	Reference			1	Reference		
Q2	239	228	1.56	1.18	2.05	0.002	1.60	1.20	2.13	0.001	1.71	1.27	2.30	3.7 × 10^−4^
Q3	243	228	1.56	1.20	2.05	0.001	1.60	1.21	2.12	0.001	1.64	1.23	2.19	0.001
Q4	270	229	1.77	1.35	2.33	4.7 × 10^−5^	1.70	1.28	2.26	2.5 × 10^−4^	1.77	1.32	2.37	1.5 × 10^−4^
Q5	232	228	1.51	1.14	1.99	0.004	1.50	1.12	1.99	0.006	1.55	1.15	2.09	0.004
					trend *p* =	0.005			trend *p* =	0.013			trend *p* =	0.013
Colorectum														
Q1	145	191	1	Reference			1	Reference			1	Reference		
Q2	171	190	1.18	0.88	1.60	0.27	1.07	0.79	1.46	0.65	1.07	0.78	1.48	0.658
Q3	223	190	1.54	1.15	2.06	0.003	1.47	1.10	1.98	0.01	1.43	1.06	1.94	0.02
Q4	234	190	1.62	1.21	2.17	0.001	1.52	1.13	2.04	0.006	1.44	1.06	1.96	0.02
Q5	177	190	1.22	0.91	1.64	0.191	1.10	0.81	1.49	0.533	1.04	0.76	1.42	0.819
					trend *p* =	0.035			trend *p* =	0.11			trend *p* =	0.30

^1^ Crude model considering matching factors (age and sex) in the conditional logistic regression model. ^2^ Model 1 further adjusted for alcohol intake, PY of smoking, and family history of subject cancer. ^3^ Model 2 further adjusted for BMI, past history of diabetes, physical activity (metabolic equivalent (MET)-hour per week), vegetable/fruit intake, beef/pork intake, and processed meat intake. ^4^ Some cases were excluded because the areal deprivation index was unknown or other (4 cases of head and neck cancer, 7 cases of esophageal cancer, 9 cases of stomach cancer, and 3 cases of colorectal cancer were excluded).

## Supplementary Materials

The following are available online at https://www.mdpi.com/2072-6694/12/11/3258/s1, Table S1: Association between educational level and risk of subject cancers by site, Table S2: Association between the quintiles of the areal deprivation index and risk of subject cancers by sites, Table S3: Association between educational level and risk of subject cancers by site stratified by age, Table S4: Association between the quintiles of the deprivation index and risk of subject cancers by sites, Figure S1: The physical distances between the residential addresses and our hospital by education level, Figure S2: The physical distances between the residential addresses and our hospital by areal deprivation index.
