# Supplementary material for: Association between Socioeconomic Status and Digestive Tract Cancers: A Case-Control Study
Source: Cancers (Basel). 2020 Nov 4;12(11):3258. doi: 10.3390/cancers12113258 (PMC7694284; doi:10.3390/cancers12113258)
Supplement: Supplementary file 1 [file cancers-12-03258-s001.pdf]

# Supplementary Materials: Association Between Socioeconomic Status and Digestive Tract Cancers: A Case-Control Study

**Yukino Kawakatsu, Yuriko N. Koyanagi, Isao Oze, Yumiko Kasugai, Hisayoshi Morioka, Rui Yamaguchi, Hidemi Ito and Keitaro Matsuo**

**Table S1.** Association between educational level and risk of subject cancers by site [1].

| Educational Level | Case | Control | Crude | | | | Model 1 | | | | Model 2 | | | |
|---|---|---|---|---|---|---|---|---|---|---|---|---|---|---|
| | | | Odds Ratio | ([95% Conf. Interval) | | *p* > z | Odds Ratio | (95% Conf. Interval) | | *p* > z | Odds Ratio | (95% Conf. Interval) | | *p* > z |
| Stomach | | | | | | | | | | | | | | |
| ≤junior high school | 122 | 83 | 1 | Reference | | | 1 | Reference | | | 1 | Reference | | |
| ≤high school | 339 | 278 | 0.81 | 0.57 | 1.17 | 0.262 | 0.82 | 0.57 | 1.18 | 0.287 | 0.78 | 0.53 | 1.15 | 0.213 |
| higher education | 326 | 426 | 0.54 | 0.37 | 0.79 | 0.002 | 0.56 | 0.38 | 0.83 | 0.004 | 0.52 | 0.34 | 0.78 | 0.002 |
| | | | | | trend *p* = | $1.7 \times 10^{-4}$ | | | trend *p* = | 0.001 | | | trend *p* = | $2.0 \times 10^{-4}$ |

[1] Model 1 and Model 2 further adjusted for *H. pylori* and atrophic gastritis.

**Table S2.** Association between the quintiles of the areal deprivation index and risk of subject cancers by sites [1].

| Quintile of ADI | Case | Control | Crude | | | | Model 1 | | | | Model 2 | | | |
|---|---|---|---|---|---|---|---|---|---|---|---|---|---|---|
| | | | Odds Ratio | (95% Conf. Interval) | | *p* > z | Odds Ratio | (95% Conf. Interval) | | *p* > z | Odds Ratio | (95% Conf. Interval) | | *p* > z |
| Stomach | | | | | | | | | | | | | | |
| Q1 | 123 | 161 | 1 | Reference | | | 1 | Reference | | | 1 | Reference | | |
| Q2 | 172 | 163 | 1.43 | 0.99 | 2.07 | 0.057 | 1.49 | 1.02 | 2.18 | 0.038 | 1.64 | 1.10 | 2.43 | 0.014 |
| Q3 | 165 | 160 | 1.23 | 0.85 | 1.77 | 0.269 | 1.25 | 0.86 | 1.81 | 0.243 | 1.28 | 0.87 | 1.89 | 0.211 |
| Q4 | 182 | 150 | 1.49 | 1.03 | 2.15 | 0.036 | 1.42 | 0.97 | 2.08 | 0.07 | 1.55 | 1.05 | 2.30 | 0.029 |
| Q5 | 151 | 159 | 1.20 | 0.83 | 1.74 | 0.327 | 1.20 | 0.82 | 1.74 | 0.348 | 1.27 | 0.86 | 1.88 | 0.224 |
| | | | | | trend *p* = | 0.392 | | | trend *p* = | 0.534 | | | trend *p* = | 0.39 |

[1] Model 1 and Model 2 further adjusted for *H. pylori* and atrophic gastritis.

**Table S3.** Association between educational level and risk of subject cancers by site stratified by age.

| Educational Level | Model 2(<60 Years Old) | | | | | | Model 2(≥60 Years Old) | | | | | | Model 2 | | | | | |
|---|---|---|---|---|---|---|---|---|---|---|---|---|---|---|---|---|---|---|
| | Case | Control | Odds Ratio | (95% Conf. Interval) | | *p* > z | Case | Control | Odds Ratio | (95% Conf. Interval) | | *p* > z | Case | Control | Odds Ratio | (95% Conf. Interval) | | *p* > z |
| Head and Neck | | | | | | | | | | | | | | | | | | |
| ≤junior high school | 19 | 15 | 1 | | | | 104 | 53 | 1 | | | | 123 | 68 | 1 | Reference | | |
| ≤high school | 100 | 77 | 1.87 | 0.78 | 4.51 | 0.161 | 128 | 138 | 0.54 | 0.34 | 0.85 | 0.009 | 228 | 215 | 0.67 | 0.43 | 1.03 | 0.069 |
| higher education | 134 | 167 | 1.12 | 0.47 | 2.64 | 0.798 | 98 | 130 | 0.47 | 0.29 | 0.77 | 0.003 | 232 | 297 | 0.43 | 0.27 | 0.68 | $3.2 \times 10^{-4}$ |

| | | | | | | | | | | | | | | | | | | |
|---|---|---|---|---|---|---|---|---|---|---|---|---|---|---|---|---|---|---|
| | | | | | trend $p$ = | 0.157 | | | | | trend $p$ = | 0.005 | | | | | trend $p$ = | $1.4 \times 10^{-4}$ |
| Esophagus | | | | | | | | | | | | | | | | | | |
| ≤junior high school | 13 | 3 | 1 | | | | | | 1 | | | | 103 | 67 | 1 | Reference | | |
| ≤high school | 70 | 58 | 0.24 | 0.03 | 1.99 | 0.184 | 90 | 64 | 0.63 | 0.38 | 1.04 | 0.070 | 193 | 175 | 0.63 | 0.35 | 1.13 | 0.121 |
| higher education | 74 | 103 | 0.13 | 0.02 | 1.08 | 0.059 | 123 | 117 | 0.64 | 0.39 | 1.06 | 0.081 | 202 | 254 | 0.64 | 0.36 | 1.15 | 0.137 |
| | | | | | trend $p$ = | 0.023 | 128 | 151 | | | trend $p$ = | 0.111 | | | | | trend $p$ = | 0.172 |
| Stomach | | | | | | | | | | | | | | | | | | |
| ≤junior high school | 29 | 16 | 1 | | | | 164 | 122 | 1 | | | | 193 | 138 | 1 | Reference | | |
| ≤high school | 170 | 129 | 0.77 | 0.39 | 1.54 | 0.464 | 311 | 286 | 0.82 | 0.61 | 1.10 | 0.184 | 481 | 415 | 0.79 | 0.60 | 1.05 | 0.105 |
| higher education | 228 | 303 | 0.44 | 0.22 | 0.88 | 0.019 | 225 | 280 | 0.63 | 0.46 | 0.86 | 0.003 | 453 | 583 | 0.52 | 0.38 | 0.69 | $9.9 \times 10^{-6}$ |
| | | | | | trend $p$ = | $1.0 \times 10^{-4}$ | | | | | | 0.003 | | | | | trend $p$ = | $5.5 \times 10^{-7}$ |
| Colorectum | | | | | | | | | | | | | | | | | | |
| ≤junior high school | 27 | 14 | 1 | | | | 139 | 93 | 1 | | | | 166 | 107 | 1 | Reference | | |
| ≤high school | 157 | 127 | 0.64 | 0.31 | 1.34 | 0.236 | 229 | 223 | 0.69 | 0.49 | 0.97 | 0.032 | 386 | 350 | 0.69 | 0.51 | 0.93 | 0.016 |
| higher education | 229 | 277 | 0.48 | 0.23 | 0.98 | 0.045 | 161 | 207 | 0.52 | 0.37 | 0.75 | $3.9 \times 10^{-4}$ | 390 | 484 | 0.52 | 0.38 | 0.71 | $2.9 \times 10^{-5}$ |
| | | | | | trend $p$ = | 0.014 | | | | | trend $p$ = | $4.1 \times 10^{-4}$ | | | | | trend $p$ = | $1.2 \times 10^{-5}$ |

**Table S4.** Association between the quintiles of the deprivation index and risk of subject cancers by sites [4].

| Quintile of ADI | Case | Control | Crude [1] | | | | Model 1 [2] | | | | Model 2 [3] | | | |
|---|---|---|---|---|---|---|---|---|---|---|---|---|---|---|
| | | | Odds Ratio | (95% Conf. Interval) | | $p > z$ | Odds Ratio | (95% Conf. Interval) | | $p > z$ | Odds Ratio | (95% Conf. Interval) | | $p > z$ |
| Head and Neck | | | | | | | | | | | | | | |
| Q1 | 98 | 116 | 1 | Reference | | | 1 | Reference | | | 1 | Reference | | |
| Q2 | 108 | 115 | 1.13 | 0.76 | 1.68 | 0.541 | 1.34 | 0.86 | 2.08 | 0.192 | 1.28 | 0.80 | 2.07 | 0.302 |
| Q3 | 116 | 116 | 1.19 | 0.81 | 1.74 | 0.370 | 1.19 | 0.78 | 1.82 | 0.415 | 1.03 | 0.65 | 1.63 | 0.907 |
| Q4 | 136 | 117 | 1.40 | 0.96 | 2.04 | 0.083 | 1.62 | 1.07 | 2.47 | 0.024 | 1.60 | 1.01 | 2.52 | 0.043 |
| Q5 | 124 | 113 | 1.30 | 0.89 | 1.89 | 0.179 | 1.47 | 0.97 | 2.24 | 0.072 | 1.53 | 0.98 | 2.41 | 0.064 |
| | | | | | trend $p$ = | 0.12 | | | trend $p$ = | 0.074 | | | trend $p$ = | 0.050 |
| Esophagus | | | | | | | | | | | | | | |
| Q1 | 71 | 99 | 1 | Reference | | | 1 | Reference | | | 1 | Reference | | |
| Q2 | 67 | 100 | 0.93 | 0.60 | 1.45 | 0.761 | 0.74 | 0.40 | 1.36 | 0.336 | 0.72 | 0.36 | 1.43 | 0.352 |
| Q3 | 95 | 98 | 1.37 | 0.90 | 2.09 | 0.137 | 1.39 | 0.81 | 2.41 | 0.234 | 1.51 | 0.80 | 2.86 | 0.203 |
| Q4 | 128 | 99 | 1.81 | 1.20 | 2.71 | 0.005 | 1.26 | 0.74 | 2.15 | 0.404 | 1.23 | 0.67 | 2.27 | 0.504 |
| Q5 | 138 | 99 | 1.95 | 1.29 | 2.95 | 0.002 | 1.50 | 0.86 | 2.62 | 0.151 | 1.77 | 0.94 | 3.34 | 0.079 |

| | | | | | | | | | | | | | | |
|---|---|---|---|---|---|---|---|---|---|---|---|---|---|---|
| | | | | | trend $p$ = | $2.5 \times 10^{-5}$ | | | trend $p$ = | 0.032 | | | trend $p$ = | 0.018 |
| Stomach | | | | | | | | | | | | | | |
| Q1 | 152 | 228 | 1 | Reference | | | 1 | Reference | | | 1 | Reference | | |
| Q2 | 238 | 228 | 1.56 | 1.18 | 2.06 | 0.002 | 1.61 | 1.20 | 2.14 | 0.001 | 1.73 | 1.28 | 2.33 | $3.3 \times 10^{-4}$ |
| Q3 | 241 | 227 | 1.56 | 1.19 | 2.05 | 0.001 | 1.60 | 1.21 | 2.12 | 0.001 | 1.64 | 1.23 | 2.20 | 0.001 |
| Q4 | 263 | 228 | 1.75 | 1.32 | 2.31 | $8.2 \times 10^{-5}$ | 1.66 | 1.25 | 2.22 | 0.001 | 1.74 | 1.29 | 2.34 | $2.7 \times 10^{-4}$ |
| Q5 | 232 | 227 | 1.53 | 1.16 | 2.03 | 0.003 | 1.54 | 1.16 | 2.06 | 0.003 | 1.61 | 1.20 | 2.17 | 0.002 |
| | | | | | trend $p$ = | 0.003 | | | trend $p$ = | 0.009 | | | trend $p$ = | 0.007 |
| Colorectum | | | | | | | | | | | | | | |
| Q1 | 145 | 189 | 1 | Reference | | | 1 | Reference | | | 1 | Reference | | |
| Q2 | 180 | 190 | 1.23 | 0.91 | 1.66 | 0.172 | 1.13 | 0.83 | 1.54 | 0.432 | 1.12 | 0.82 | 1.54 | 0.472 |
| Q3 | 212 | 187 | 1.47 | 1.10 | 1.96 | 0.01 | 1.40 | 1.04 | 1.88 | 0.027 | 1.37 | 1.01 | 1.86 | 0.044 |
| Q4 | 233 | 189 | 1.60 | 1.20 | 2.14 | 0.001 | 1.51 | 1.12 | 2.03 | 0.007 | 1.43 | 1.05 | 1.95 | 0.024 |
| Q5 | 175 | 188 | 1.21 | 0.90 | 1.62 | 0.217 | 1.09 | 0.80 | 1.48 | 0.584 | 1.02 | 0.75 | 1.41 | 0.880 |
| | | | | | trend $p$ = | 0.075 | | | trend $p$ = | 0.209 | | | trend $p$ = | 0.463 |

[1] Crude model considering matching factors (age and sex) in the conditional logistic regression model. [2] Model 1 further adjusted for alcohol intake, PY of smoking, and family history of subject cancer. [3] Model 2 further adjusted for BMI, past history of diabetes, physical activity (metabolic equivalent (MET)-per week), vegetable/fruit intake, beef/pork intake, and processed meat intake. [4] Some cases were excluded, because the areal deprivation index was unknown or unstable (13 cases of head and neck cancer, 8 cases of esophageal cancer, 23 cases of stomach cancer, and 15 cases of colorectal cancer were excluded).

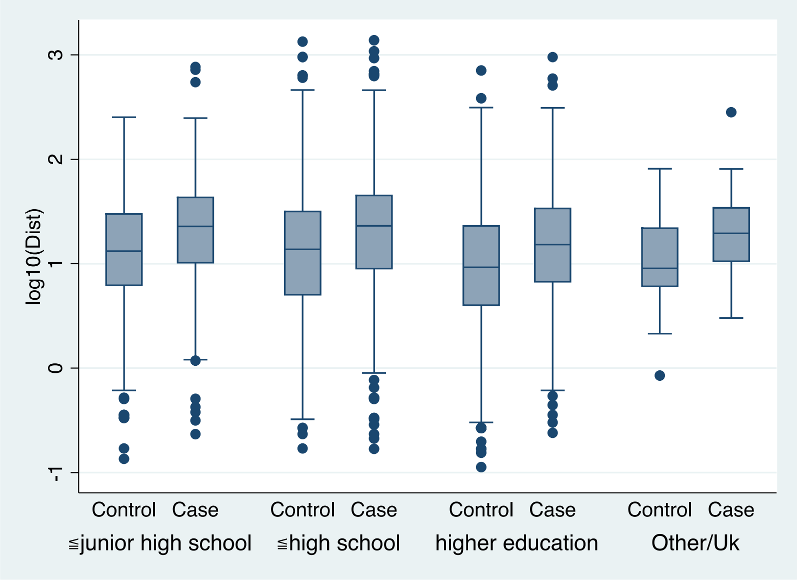


**Figure S1.** The physical distances between the residential addresses and our hospital by education level.

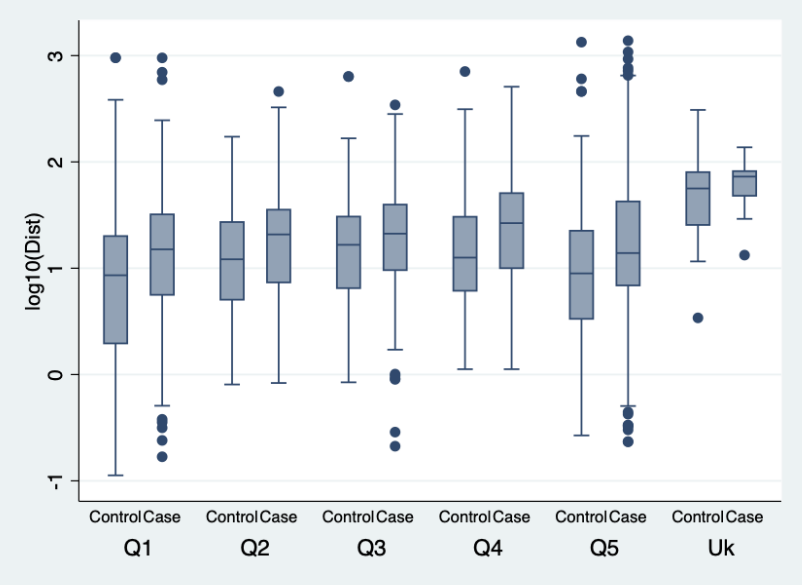


**Figure S2.** The physical distances between the residential addresses and our hospital by areal deprivation index.

**Publisher's Note:** MDPI stays neutral with regard to jurisdictional claims in published maps and institutional affiliations.

© 2020 by the authors. Licensee MDPI, Basel, Switzerland. This article is an open access article distributed under the terms and conditions of the Creative Commons Attribution (CC BY) license (http://creativecommons.org/licenses/by/4.0/).
